# The Use of Terahertz Computed Tomography and Time Domain Spectroscopy to Evaluate Symmetry in 3D Printed Parts

**DOI:** 10.3390/polym16233296

**Published:** 2024-11-26

**Authors:** Dolores Termini, John Federici, Ian Gatley, Louis Rizzo

**Affiliations:** 1 Department of Physics, New Jersey Institute of Technology, 323 Dr. M.L.King. Jr. Blvd., Newark, NJ 07102, USA; john.federici@njit.edu (J.F.); ian.gatley@njit.edu (I.G.); 2 U.S. Army DEVCOM Armaments Center, Picatinny Arsenal, Wharton, NJ 07885, USA; louis.j.rizzo14.civ@army.mil

**Keywords:** terahertz, 3D printing, additive manufacturing, non-destructive evaluation, imaging, computed tomography

## Abstract

3D printing has become essential to many fields for its low-cost production and rapid prototyping abilities. As 3D printing becomes an alternative manufacturing tool, developing methods to non-destructively evaluate defects for quality control is essential. This study integrates the non-destructive terahertz (THz) analysis methods of terahertz time-domain spectroscopy (THz-TDS) and terahertz computed tomography (THz CT) to image and assess 3D printed resin structures for defects. The terahertz images were reconstructed using MATLAB, and the rotational symmetry of various structures before and after the introduction of defects was evaluated by calculating the mean squared deviation (MSD), which served as a symmetry parameter to indicate the presence of defects. Structures A and B had MSD values that were at least three standard deviations larger after introducing defects to their structures, showing a significant change in symmetry and indicating the existence of defects. Similarly, in structure C, blockages in parts made with different post-cures were identified based on the increase in MSD values for those slices. For structure D, the presence of a defect increased the MSD value by 14%. The results of this study verify that the MSD calculated for the rotational symmetry of the structures was greater when defects were present, accurately reflecting the anticipated breaks in symmetry. This paper demonstrates that terahertz imaging, combined with MSD analysis, is a viable procedure to identify and quantify defects in rotationally symmetric 3D printed structures.

## 1. Introduction

Terahertz (THz) radiation, which falls between infrared and microwaves on the electromagnetic spectrum, has become a popular tool for analysis over the last three decades. It is excellent for non-contact, non-destructive evaluation (NDE) when transmitted through various non-conductive materials using terahertz time-domain spectroscopy (THz-TDS) and terahertz computed tomography (THz CT). In THz-TDS, the terahertz beam that is partially transmitted through a material can provide insight into structural and optical properties based on the frequency and timing information contained in the waveform [1]. THz-TDS taken at multiple points in a grid formation can result in an image that shows spatial variations in structural features or non-uniformity of a material based on spatial variations in the material’s terahertz-specific properties. Applications for THz-TDS include skin tissue imaging, breast cancer imaging, forestry, and security scanning [2,3,4]. Terahertz spectroscopy has been employed more recently for biomedical uses, like identifying more types of cancer, including digestive, cervical, and prostate cancers [5].

THz CT is a methodology for creating a 3D reconstruction of a part or material by acquiring a THz-TDS image, rotating the structure a certain amount, and then repeating this process for multiple rotational increments. The THz-TDS measurements are then processed to create a stack of images that can be made into a 3D reconstruction suitable for locating defects in a material [1]. THz CT has a similar procedure to X-ray computed tomography (X-ray CT), which is commonly used in the medical field, but THz CT has been gaining traction in other fields since the longer wavelength interacts with materials differently than X-rays. While X-rays have better spatial resolution, terahertz waves can identify and image materials normally transparent to X-rays and are non-destructive to many materials [6]. The variety of applications for THz CT include defect detection in polymers reinforced with glass fibers, imaging and weight determination of sunflower seed kernels, and imaging of historical artifacts like clay pots [7,8,9].

This study investigates the application of terahertz imaging for symmetric 3D printed structures. 3D printing and additive manufacturing (AM) have become popular since they are a cheap, fast, and effective way of creating parts. It can be used for applications such as creating prototypes, medical models, medication, and bone tissue [10,11]. In fused deposition modeling (FDM), one of the most common types of 3D printing, a part is built by repeatedly depositing layers of extruded thermoplastic material in a desired pattern until the part is fully formed [12]. FDM printing uses thermoplastics like PLA, but stereolithography (SLA) or vat polymerization printing uses other materials, like resin, to create parts with finer and more complicated features due to the small layer height [13]. Resin SLA 3D printing works by applying a thin layer of liquid resin in a specific pattern and using an ultraviolet laser to harden the resin between each layer application [14]. While 3D printing has significantly evolved since its early stages, it is still easy for simple printed structures to “fail”, meaning the printer stops printing in the middle or there is non-uniformity in the plastic. In the case of 3D resin printing, some leading issues are trapped uncured resin, resin contamination, or delamination of layers [13,15]. 

Previously, THz-TDS was used to analyze the terahertz absorption and properties of materials used in FDM for various purposes, including improving components in terahertz systems [16]. There are several studies on using additively manufactured lenses, fibers, and metamaterials for terahertz imaging [17,18,19]. AM allows for much advancement in terahertz systems components due to easy prototyping and customization [20,21]. However, there are notably fewer sources that use terahertz characterization for structures created with AM. Terahertz imaging and evaluation have been used to investigate losses in 3D printed waveguides and surface features of additively manufactured components [22,23]. In the past, THz-TDS and THz CT were used to inspect infill patterns and polymer structures created with 3D printing, but it is not common practice [24,25]. With the push for 3D printing to become a large-scale manufacturing process, an emerging testing methodology is needed for automated quality control to detect defects within a reasonable threshold. There is significant potential for terahertz non-destructive evaluation to investigate misprints and inconsistencies in structures created with AM.

Within AM, parts must adhere to many tolerances and standards. While traditional tolerance analysis uses established standards like ISO 1101 for geometrical and situational tolerances, our method employs mean squared deviation (MSD) to evaluate symmetry and detect defects in 3D printed structures [26]. MSD symmetry analysis, derived from terahertz imaging data, quantifies rotational symmetry deviations and enables non-contact, non-destructive evaluation (NDE). This technique uncovers internal inconsistencies or asymmetries in complex geometries, complementing conventional dimensional tolerance methods. Although MSD is not a replacement for ISO-standard tolerance analysis, it offers valuable insights for structural integrity, particularly in NDE contexts.

This study aims to utilize THz-TDS and THz CT to investigate various structures created with SLA 3D printing to identify and quantify defects, specifically in structures that should have a high degree of rotational symmetry. In this work, four rotationally symmetric structures were 3D printed without defects. For each structure, defects expected to disrupt the rotational symmetry were introduced using dental wax, super glue, or different curing methods. The diversity of structures and defects tested shows that the imaging techniques in this study are greatly versatile in identifying defects in samples with expected rotational symmetry. THz CT was conducted on three structures, and THZ-TDS was conducted on one structure that was too thick for terahertz THz CT. The terahertz images were processed using MATLAB to create 3D reconstructions. From the reconstructions, the defects were identified using the mean square deviation to compare the image after each rotation. Symmetry analysis was successfully used for defect identification with both THz CT and THZ-TDS.

## 2. Materials and Methods

All the structures were SLA printed using a Phrozen Sonic 4K (2022 model) with CPS PR221 glitter resin. Each structure was printed from an STL file uploaded to the ChiTuBox v1.8.1 slicer to slice the design and then loaded into the printer. Each standard resin layer was exposed to a UV laser for 3 s, and the initial layers had an exposure time of 18 s to ensure the structure’s base was stable. Details regarding equipment, printing parameters, and software are listed in Table 1. The printing parameters were chosen based on the optimal settings for the resin, structure design, and printer used 3D printed parts.

**Figure 1:**
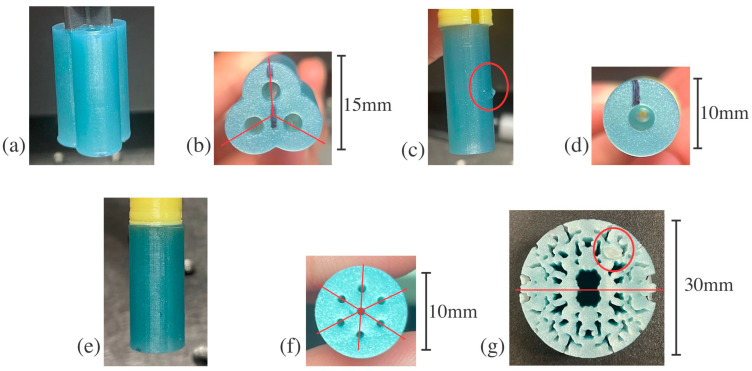
3D printed structures with various rotational symmetries: (**a**) structure A side profile, (**b**) bottom of structure A with holes that propagate through the structure in the y-direction and trifold symmetry marked with lines of symmetry, (**c**) structure B side profile with external 2 mm defect circled, (**d**) bottom of structure B with the hole that propagates through the structure in the y-direction, (**e**) structure C side profile, (**f**) bottom of structure C with the seven holes that propagates through the structure in the y-direction and six-fold symmetry marked with lines of symmetry, (**g**) structure D front profile with an external 4 mm dental wax defect circled and twofold symmetry marked with a horizontal line.

The structures were designed to have different sizes and shapes to show the versatility of terahertz imaging in determining the presence of defects and lack of symmetry. The structures are designs engineered by the U.S. Army Combat Capabilities Development Command (DEVCOM) Armaments Center with clear lines of various rotational symmetries, shapes, and sizes to test the versatility of THz CT and THz-TDS, as seen in Figure 1. Defects of various sizes were created after the printing process was completed, except for the defect in Figure 1d, which was a fault of the printer itself. After printing, different samples with structure C (as shown in Figure 1e,f) were either cured with UV light for 30 min, cured in a furnace for 30 min at 50 °C in the Formlabs Form Cure L, or had no post-cure, like all the other structures that were tested. This could lead to different terahertz indices of refraction or uncured resin within the structure, both of which act as types of defects, as seen later in this paper.

The TeraMetrix T-Ray 5000 Series Intelligent Terahertz Control Unit and Instrumentation was used to perform all terahertz measurements and imaging. THz-TDS is conducted at multiple points in a grid pattern to form an image with a pixel size of 0.2 mm and 120 waveforms per pixel averaging. The sample is mounted on an XY translational stage between a terahertz transmitter and receiver since THz-TDS and THz CT will be done in transmission for this research. After the time domain signal of the terahertz electric field is received, the information is converted to the frequency domain using a fast Fourier transform [1]. In THz CT, a THZ-TDS image is taken, then the structure is rotated 5 degrees, and another THZ-TDS image is acquired. 2D images were acquired until the structure had rotated 360 degrees for 72 THz-TDS images that were later reconstructed into a 3D model.

The structures examined here were investigated with THz-TDS or THz CT, but not both. The terahertz imaging technique for each structure was determined based on its thickness and shape. THz-TDS is best when the terahertz signal is perpendicular to a planar surface. Structures A, B, and C all have planar surfaces in the orientation in Figure 1b,d,f but are too thick for adequate terahertz signal transmission in that direction. The structure surfaces are curved in the orientations in Figure 1a,c,e. At the boundary between air and the material, the beam will refract at an angle determined by the material’s real index of refraction and the angle at which the beam enters the material. If the index of refraction of the structure were too big compared to that of air, then the angle of refraction would be too large, and the detector would not receive the beam. In addition, if the surface structure is curved, like structures A, B, and C, the beam will be refracted at a different degree at each point since the entry angle constantly changes, leading to a distorted image and significant losses [6].

**Figure 2:**
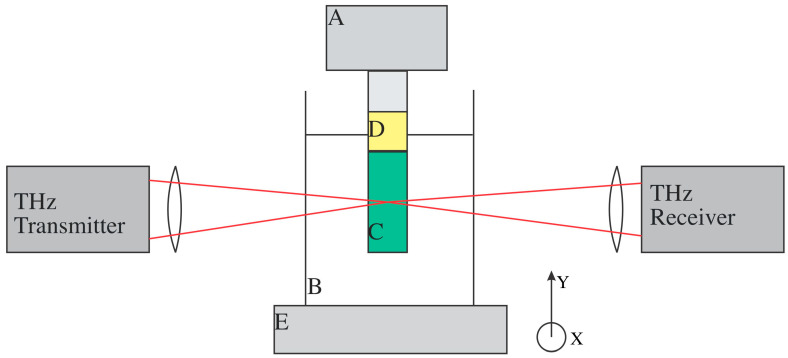
Schematic of THz CT apparatus layout: (A) The upside-down 360° rotational stage is controlled by a LabView program, (B) A 3D printed container filled with mineral oil to provide a planar surface, (C) The structure is glued to a (D) 3D printed mount that allows mineral oil to flow through the holes. The mount is screwed into a most that is screwed into the 360° rotational stage, (E) The XY stage between the terahertz transmitter and receiver that the THz CT apparatus is mounted upon. From this view, the x-direction of the translational stage is in and out of the page, while the y-direction is up and down.

THz-TDS images can be reconstructed with THz CT. To alleviate the issue of signal loss due to refraction at the air/structure boundaries, structures A, B, and C were submerged in a rectangular tank of mineral oil, seen in Figure 2, to create a perpendicular, planar surface for the terahertz beam to enter after traveling through the air. Samples with structure C were sonicated in mineral oil for 30 min before starting the THz CT measurement since the holes were significantly smaller than other structures, and the surface chemistry could be different due to the different UV and thermal post-cures. The mineral oil has a terahertz real index of refraction in the range of ~1.47, which was similar to that of the structures, so the angle of refraction after going through the structure was not so large that the signal did not reach the detector [27]. However, the refractive index difference between the mineral oil and 3D printed material was large enough to distinguish features in the structures. The THz-TDS images taken during THz CT for structures C, D, and E had a 0.2 mm pixel size and 120 waveforms per pixel averaging. The THz-TDS images were generated based on the natural log of the transmitted electric field in the 0.4 to 2 THz spectral range. Each THz-TDS image is seen as a projection at the angle at which it was taken. In MATLAB, the iradon function was then used to create horizontal slices, contained in .txt files, from the THz-TDS image projections, which can then be stacked to create a reconstruction [28]. The horizontal slices are expected to resemble structures A, B, and C in Figure 1b,d,f.

Structure D, orientation shown in Figure 1g, was measured with THz-TDS before and after the introduction of defects. THz-TDS was chosen as the imaging method for these structures since they are 7 mm thick in this orientation, allowing for adequate terahertz signal transmission and providing a planar perpendicular surface to the terahertz beam. It is important to note that THz CT was not conducted for structure D, and it was not submerged in mineral oil for THz-TDS measurements since its features are small, and it would be challenging to ensure that the mineral oil would permeate all the hole-like features. For optimal contrast, the THz-TDS images were generated based on the natural log of the transmitted electric field in the 0.6 to 2 THz range for structure D. The expected THz-TDS image should have features very similar to those of the structure.

MATLAB was used for all analyses, and FIJI was initially used to reconstruct the THz CT measurement for structures A, B, and C. FIJI is a Java-based image processing and analysis software. In MATLAB, the iradon function creates horizontal slices of the structure and stores those slices in .txt files. The slices are then stacked to create the reconstruction in the volume viewer in FIJI. It is important to note that terahertz imaging does not provide an exact reconstruction of a structure. However, the terahertz images can show the features of a structure and if a defect is present.

Defects were determined by evaluating the rotational symmetry in each slice using MATLAB. Each pixel in the slice represents the natural log of the transmitted electric field in the frequency ranges of 0.4 to 2 THz for structures A, B, and C and 0.6 to 2 THz for structure D. The statistical data processing to determine the symmetry of each slice is as follows: the mean squared deviation (MSD) was calculated between the slice in its original orientation and the slice after it was rotated a predetermined number of degrees. The number of degrees rotated is based on the type of rotational symmetry for that structure. MSD is a method to calculate the sum of the squared differences between expected and measured values [29]. In this case, the pixel values of the slice in its original orientation are the “expected values”. The pixel values after the slice has been rotated are the “measured values”. In contrast to the base and reference levels utilized in ISO 1101, MSD symmetry is conducted by comparing spatially mapped terahertz data with an idealized symmetrical model. This methodology facilitates the identification of asymmetries in materials and structures that would be difficult to evaluate using conventional tactile or dimensional techniques, thereby rendering it particularly advantageous for NDE in polymers or internal 3D printed structures. When there is a high degree of symmetry with few defects, a lower MSD calculation is anticipated, but when defects exist, a higher MSD calculation is expected. The exact process was applied to the THz-TDS images, stored in .csv files, taken for structure D. The type of rotational symmetry for each structure was determined based on the expected pattern. When rotating the slices or terahertz images of the structures, it is critical to rotate about the centroid of the structure itself rather than the center of the .csv or .txt file. Different centroid determination methods were employed to determine which was best to locate the centroid of each structure. Table 2 states the number of folds of symmetry, rotation step, and methods for centroid determination used in the MATLAB code that used MSD to quantify the symmetry for each structure.

## 3. Results and Discussion

**Figure 3:**
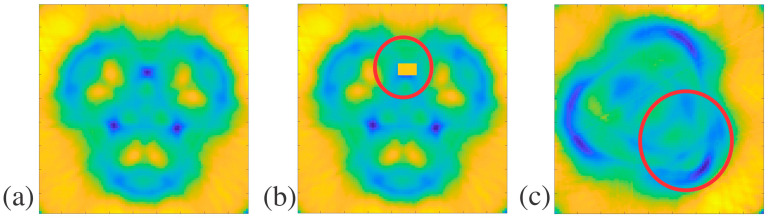
Structure A THz CT slice reconstructions in MATLAB: (**a**) structure A slice without defects, (**b**) structure A slice with an artificial defect indicated by the red circle, and (**c**) structure A slice with one hole filled with air indicated by the red circle.

**Figure 4:**
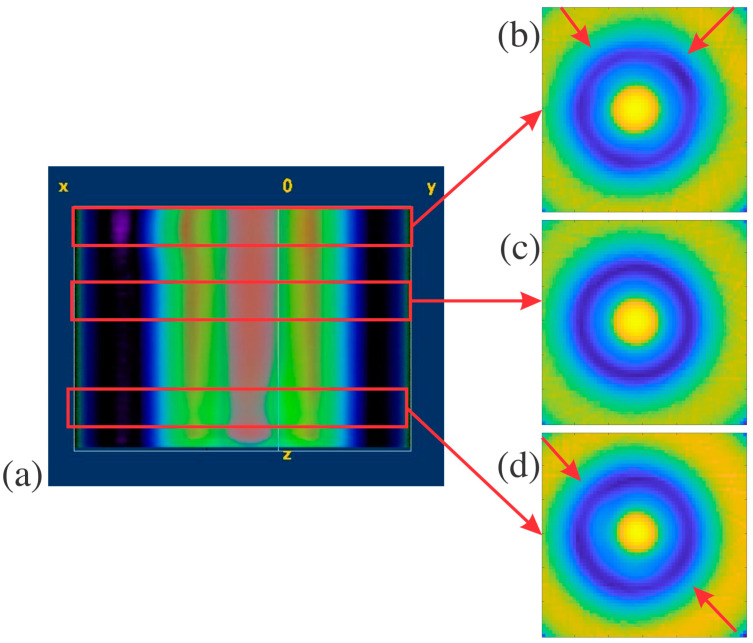
Structure B THz CT slice reconstructions in FIJI and MATLAB: (**a**) shows a FIJI reconstruction of structure B with internal and external defects, used to identify the location of the defects, with red arrows pointing to different cross-sectional areas of the reconstruction, (**b**) MATLAB plot of structure B with an external defect and inconsistencies pointed out by the red arrows, (**c**) MATLAB plot of a structure B slice without defects, and (**d**) MATLAB plot of a structure B with an internal defect and inconsistencies pointed out by the red arrows.

**Figure 5:**
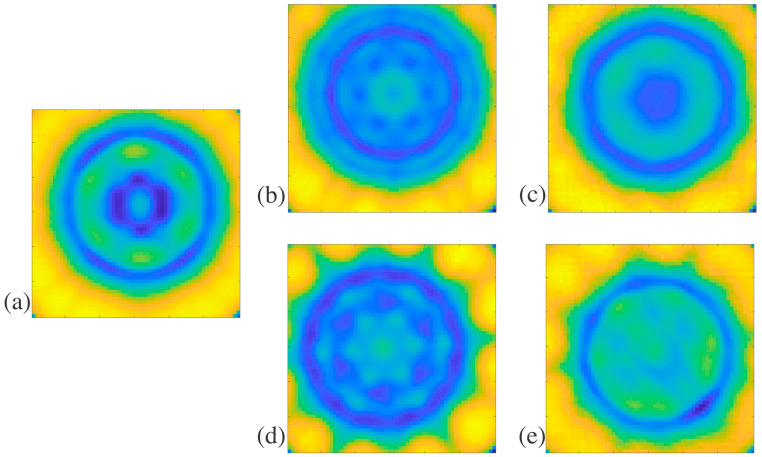
Structure C THz CT slice reconstructions in MATLAB: (**a**) no post-cure slices without defects, (**b**) thermal post-cure slice without defects, (**c**) thermal post-cure slice with blockages, (**d**) UV post-cure slice without defects, and (**e**) UV post-cure slice with blockages.

**Figure 6:**
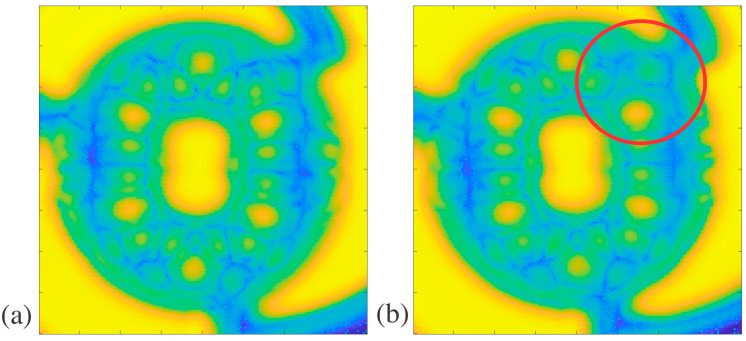
Structure D THz-TDS plots in MATLAB: (**a**) structure D front profile and (**b**) structure D with an external 4 mm defect circled in red.

Arrays of the structures were plotted in MATLAB using color mapping to visualize the patterns and breaks in the patterns due to defects, as seen in Figure 3, Figure 4, Figure 5 and Figure 6. While structure D was imaged with THz-TDS, structures A, B, and C were imaged with THz CT and then went through post-measurement processing to create the horizontal slices of each sample, as seen in Figure 3, Figure 4 and Figure 5. For structure A, a sample free of defects was measured with THz CT, and then the average trifold symmetry MSD of those slices was calculated and used to determine the standard deviation, as seen in Table 3. This was also calculated for structure B except with complete radial instead of tri-fold symmetry.

Two different defects were introduced to structure A. The first was an artificial defect created by changing the values of pixels in the circled region of Figure 3b. The second defect was created by filling one of the three holes in the structure with air in the circled region of Figure 3c. Since there would be a large difference in the index of refraction between the sample and the air within the hole, this should significantly alter how much of the terahertz beam was captured by the receiver, creating a lack of symmetry in the terahertz image, as shown in Table 3. The MSD values for the artificial defect and hole filled with air defect in structure A were 0.767 and 1.875, respectively. The average trifold symmetry MSD value for structure A was 0.357, with a standard deviation of 0.042. The MSD values of slices with either defect were more than three standard deviations larger than the average without defects. This shows that defects that significantly alter the symmetry of the sample can be detected with THz CT reconstruction and then quantified using MSD. When structure A had apparent defects, the MSD was higher than the average slice without any defects.

Structure B is less complicated than others in this paper, with a single hole propagating through the middle as its defining feature. The slices of structure B were evaluated for total radial symmetry, meaning that if the slice was rotated any number of degrees about the centroid, the MSD value should be minimal. Twenty-five slices of structure B were evaluated for radial symmetry, and their MSD values were calculated to retrieve an average of 2.07 and a standard deviation of 0.13. Super glue was used to create defects on the outside and inside of structure B, as pointed out in Figure 1c. These defects are difficult to see in the individual reconstructed horizontal slices for structure B. Instead, the whole sample was reconstructed in FIJI, as seen in Figure 4a, to determine if the defects were readily apparent in the 3D rendering. With the slices stacked on each other, it was clear that there was non-uniformity in the structure. This is further seen in the MATLAB plots of slices with defects in Figure 4b,d, compared to the plot without any defects in Figure 4c. This is most likely caused by the wavelength size in terahertz imaging, which limits resolution. The defects may have been on the order of magnitude of the wavelength, which caused the inconsistencies in Figure 4b,d instead of replicating the exact shape of the defects. The MSD values for reconstructed slices with external and internal defects in structure B are 2.63 and 2.73, respectively. These values are larger than the average for structure B by at least three standard deviations, showing a significant break in the radial symmetry. The minor defects introduced to structure B can be measured with THz CT and then detected by symmetry investigation since they had high MSD values.

Structure C is the most complicated structure measured by THz CT here since the outside diameter is similar to structure B but with seven small holes propagating through the sample, each a millimeter in diameter. These small features are on the order of magnitude of terahertz waves, and it was unclear if they would be detected with THz CT. The seven holes were arranged to indicate six-fold symmetry, as shown in Figure 1f, which shows the bottom view of structure C. In addition, samples with structure C were treated with either no post-cure, like structures a, b, and d, a thermal post-cure, or a UV post-cure. Figure 5a,b,d show the slice reconstructions for structure C with no post-cure, thermal post-cure, and UV post-cure, respectively. The post-cures affected the uniformity and index of refraction of the resin, affecting how the terahertz beam refracts through the samples.

In addition to exhibiting non-uniformities attributable to the different cures, samples with structure C were also found to have other physical defects. The holes should have propagated through the length of the structure, but this was not the case. This was checked by inserting a needle into each hole to check its depth. A suspected blockage was identified when a needle could not go through a hole in the structure without resistance. The blockages were suspected to be uncured resin trapped within the structure, where the printer accidentally filled the holes. Figure 5c,e shows the reconstructed slices where blockages are present.

Separate samples were prepared where slabs of the printed material were treated with different cures. Slabs of the printed material with different post-cures and the completely uncured resin were tested for differences in index of refraction. The refractive indices for the uncured resin, no post-cure slab, thermal post-cure slab, and UV post-cure slab were 1.52, 1.67, 1.64, and 1.68, respectively. Since the slabs with different cures do not have the same index of refractions, the cures can also be seen as defects. As a result of the blockages in samples with structure C, only ten reconstructed slices were completely free of physical defects for each type of post-cure. Average MSD values for the six-fold symmetry of 10 blockage-free slices and a standard deviation were calculated for the samples with no post-cure, thermal post-cure, and UV-post-cure in Table 4. The different cures had very different average MSD values, showing that the cures can be distinguished based on the MSD value and the different indices of refraction previously mentioned. In addition to distinguishing the samples with different cures based on their average MSD, in the samples containing blockages with thermal and UV post-cures, the MSD values were 2.429 and 1.471. The MSD values in both cases are larger than the average for their respective cures by at least two standard deviations, as seen in Table 4. This indicates that there is a break in symmetry and confirms that there are defects in the selected reconstructed slices with suspected blockages.

Structure D was measured using THz-TDS since it had a planar surface in the orientation seen in Figure 1g and had too many tiny crevices that mineral oil may not be able to fill if measured with THz CT. The results of the THz-TDS for structure D can be seen as MATLAB plots in Figure 6a,b and as MSD values in Table 5. The defect seen in Figure 6b was created using dental wax. Two-fold symmetry was evaluated for structure D. Before defect introduction, the twofold symmetry of structure D was evaluated to have an MSD of 2.182, as stated in Table 5. After introducing the dental wax defect in the upper right corner of the structure, the MSD was 2.497. In comparison, the MSD of structure D with a defect was higher than that of structure D without a defect by 0.31. Although there is no error range based on the standard deviation for this structure, the defect is apparent in the reconstructions, and the MSD is over 14% larger than that of the no-defect sample. This strongly suggests that the symmetry was affected by introducing a defect, showing that defect detection is possible with THz-TDS.

This paper demonstrates the potential of MSD analysis to detect structural asymmetries in 3D printed parts independent of traditional tolerances set by ISO 1101 [26]. ISO standards specify tolerances within an external reference frame, while MSD analysis non-invasively evaluates symmetry within a structure’s volume, even with inaccessible internal features. Future work could correlate MSD thresholds with ISO tolerance levels, enabling a hybrid approach that includes terahertz-based analysis and traditional tolerancing. Research could focus on developing a framework that links MSD values with tolerance zones by creating calibration tests on standardized 3D printed samples with known tolerance deviations. These studies would assist practitioners in interpreting MSD results within traditional tolerance limits, promoting wider use in mechanical engineering and additive manufacturing quality control. Additionally, exploring diverse post-processing techniques, materials, and geometrical complexities could yield data on how MSD values vary with different manufacturing variables, enhancing the method’s applicability.

Although terahertz imaging has become popular as a non-destructive evaluation technique, it is not frequently used for defect detection within 3D printed structures. This work builds upon earlier results that identify internal structures within 3D printed parts, such as the infill pattern or different substances within the structures [25,30,31]. While [25] concentrates on the application of THz-TDS in both transmission and reflection to identify the infill pattern of the components, this study only employs THz-TDS and THz CT in transmission to evaluate thicker structures with more robust, intricate features. Another study examined the use of THz CT to measure volumetric data pertaining to 3D printed structures; however, the research contained within this paper extends upon this by employing symmetry as the criterion for evaluating defects in components exhibiting anticipated symmetry [24]. This paper expands upon the findings in [30] by using THz CT, to recognize the parts that have structure C in this paper made with different post-cures and trapped uncured resin due to their contrasting reconstructions. Although one study employs long-term memory classification networks to analyze THz-TDS measurements of materials and voids in polymers, this evaluation method is excessive for assessing rotational symmetries and identifying defects [31]. However, it may serve as a valuable approach in the future to further validate the presence of uncured resin within 3D printed parts, like in structure C. The resolution of THz CT exhibits limitations, prompting research into enhanced analytical methods, including deep learning or advancements in metal artifact suppression. Nonetheless, this study emphasizes using rotational symmetry exclusively for defect identification, rendering complex algorithms unnecessary [32,33].

## 4. Conclusions

In this paper, multiple 3D printed structures were investigated with terahertz imaging techniques before and after defect introduction. Samples with curved sides—structures A, B, and C—were measured using THz CT, while samples with large planar surfaces—structure D—were measured with THz-TDS. MATLAB was used to reconstruct each sample and measure the rotational symmetry using the mean squared deviation (MSD) to quantify defects that disrupt the symmetry.

In structures analyzed through THz CT, the MSD values of multiple defect-free slices were averaged to calculate a standard deviation, thereby determining a standard for how large the MSD value must be to indicate defects. In slices with defects, structures A and B had MSD values three standard deviations larger than those of the defect-free slices. The regions in the structure C samples with suspected uncured resin blockages showed MSD values two standard deviations greater than those of the defect-free areas in the structure. For structure C, the difference in the refractive indices and MSD values could identify variations in the post-curing process in addition to identifying defects. The results for structures A, B, and C from Section 3 show success in defect identification of defects and different post-cures. The samples measured with THz CT and reconstructed in MATLAB indicated that an analysis of rotational symmetry using MSD could be employed for defect detection.

In structure D, analyzed with THz-TDS due to its shape, the MSD value increased by 14% following the introduction of a defect. This observation indicates that planar structures, characterized by restricted imaging orientations, can be evaluated for defects with terahertz-based symmetry analysis. For structure D, an MSD average of defect-free slices was not calculated because only one THz-TDS image can be produced for a sample in the measured orientation, unlike the multiple slices generated for a THz CT measurement of the same sample. As a result, more defect-free samples with structure D, measured by THz-TDS, need to be measured to calculate an acceptable standard deviation of the MSD. Structures measured with THz-TDS alone show promise in defect detection with rotational symmetry but need more repeated measurements to prove the consistency of this investigation method.

Moreover, additional research could focus on categorizing defects in more 3D printed components with diverse structures. Future studies may explore translating MSD symmetry measurements into parameters corresponding to widely recognized standards, such as ISO standards, particularly for applications in additive manufacturing. Developing criteria that align with established standards could bridge this NDE method with conventional technical drawing tolerances, facilitating broader adoption among mechanical engineers and quality control professionals. The findings within this study collectively validate the use of THz imaging and symmetry analysis to evaluate 3D printed parts non-destructively. Although further exploration is required to enhance the methodology and broaden the sample size, this study provides a foundation for utilizing rotational symmetry as a key criterion in defect detection. Future research should aim to automate defect identification and enhance the resolution of THz imaging to augment its applicability in industrial settings.

## Figures and Tables

**Table 1:** Details for equipment and software.

Equipment and Software Parameters	
Printer Model	Phrozen Sonic 4K 2022 (Phrozen, Hsinchu City, Taiwan)
Resin	CPS PR221
Slicing Software	ChiTuBox v1.8.1 (ChiTuBox, Shenzhen, China)
UV laser layer exposure time	3 s
UV post-cure exposure time	30 min
Furnace Model	Formlabs Form Cure L (Formlabs, Somerville, MA, USA)
Thermal post-cure exposure time	30 min
Thermal post-cure exposure temperature	50 °C
Terahertz equipment and software	TeraMetrix T-Ray 5000 Series Intelligent Terahertz Control Unit and Instrumentation (Luna Innovations, Roanoke, VA, USA)
3D reconstruction software	MATLAB, FIJI

**Table 2:** Symmetry Code Parameters for Each Structure.

Structure	Folds of Symmetry	Array Rotation Step	Centroid Determination Method
A	3	120°	Outer Edge Detection
B	360	1°	Outer Edge Detection
C	6	60°	Largest Connected Area
D	2	180°	Manual Entry

**Table 3:** Average Symmetry MSD vs. Symmetry MSD with Defects for Structures A and B.

Structure	MSD (10^−6^)	Structure	MSD (10^−6^)
A (no defect)	0.357 ± 0.042	B (no defect)	2.07 ± 0.13
A (artificial defect)	0.767	B (internal defect)	2.73
A (hole filled with air)	1.875	B (external defect)	2.63

**Table 4:** Average Symmetry MSD vs. Symmetry MSD with Defects for Structure C with different post-cures.

Structure C Post-Cure	Average MSD (10^−6^)	MSD with Suspected Defect (10^−6^)
No post-cure	3.947 ± 0.078	-
Thermal post-cure	1.929 ± 0.055	2.429
UV post-cure	1.324 ± 0.062	1.471

**Table 5:** Average Symmetry MSD vs. Symmetry MSD with Defects for Structure D.

Structure	MSD Without Defect (10^−3^)	MSD with Defect (10^−3^)	Difference Between MSD with and Without DEFECT (10^−3^)
D	2.182	2.497	0.31

## Data Availability

Data are contained within the article. Raw data can be made available upon contacting Dolores Termini at email dat46@njit.

## References

[1] Wang S., Zhang X.-C. (2004). Pulsed Terahertz Tomography. J. Phys. D Appl. Phys..

[2] Guillet J.P., Recur B., Frederique L., Bousquet B., Canioni L., Manek-Hönninger I., Desbarats P., Mounaix P. (2014). Review of Terahertz Tomography Techniques. J. Infrared Millim. Terahertz Waves.

[3] Yang X., Zhao X., Yang K., Liu Y., Liu Y., Fu W., Luo Y. (2016). Biomedical Applications of Terahertz Spectroscopy and Imaging. Trends Biotechnol..

[4] Wang Y., Gao R., Ma L., Kang K., Wang C., Guo Y., Ge X. (2024). Analysis of the Application Status of Terahertz Technology in Forestry. Eur. J. Wood Wood Prod..

[5] Gezimati M., Singh G. (2024). Terahertz Imaging Technology for Localization of Cancer Tumours: A Technical Review. Multimed. Tools Appl..

[6] Duhant A., Triki M., Strauss O. (2019). Terahertz Differential Computed Tomography: A Relevant Nondestructive Inspection Application. J. Infrared Millim. Terahertz Waves.

[7] Wu Q.Y.S., Zhang N., Lim V., Zhang L., Zhong Y., Russell B., Ke L. (2024). Detection of a Glass Fiber-Reinforced Polymer with Defects by Terahertz Computed Tomography. ChemPhysMater.

[8] Lei T., Sun D.-W. (2023). Introducing the THz Time Domain CT System for Evaluating Kernel Weight and Plumpness of Sunflower Seed. J. Food Meas. Charact..

[9] Caumes J.-P., Younus A., Salort S., Chassagne B., Recur B., Ziéglé A., Dautant A., Abraham E. (2011). Terahertz Tomographic Imaging of XVIIIth Dynasty Egyptian Sealed Pottery. Appl. Opt..

[10] Berman B. (2012). 3-D Printing: The New Industrial Revolution. Bus. Horiz..

[11] Bose S., Vahabzadeh S., Bandyopadhyay A. (2013). Bone Tissue Engineering Using 3D Printing. Mater. Today.

[12] Akbaş O.E., Hıra O., Hervan S.Z., Samankan S., Altınkaynak A. (2019). Dimensional Accuracy of FDM-Printed Polymer Parts. Rapid Prototyp. J..

[13] Taormina G., Sciancalepore C., Messori M., Bondioli F. (2018). 3D Printing Processes for Photocurable Polymeric Materials: Technologies, Materials, and Future Trends. J. Appl. Biomater. Funct. Mater..

[14] Dhanunjayarao B.N., Naidu N.V.S., Kumar R.S., Phaneendra Y., Sateesh B., Olajide J.L., Sadiku E.R., Kharissova O.V., Martínez L.M.T., Kharisov B.I. (2020). 3D Printing of Fiber Reinforced Polymer Nanocomposites: Additive Manufacturing. Handbook of Nanomaterials and Nanocomposites for Energy and Environmental Applications.

[15] Dhanunjayarao B.N., Swamy Naidu N.V. (2022). Assessment of Dimensional Accuracy of 3D Printed Part Using Resin 3D Printing Technique. Mater. Today Proc..

[16] Alfattani R. (2022). Comprehensive Study on Materials Used in Different Types of Additive Manufacturing and Their Applications. Int. J. Math. Eng. Manag. Sci..

[17] Xu G., Skorobogatiy M. (2023). 3D Printing Technique and Its Application in the Fabrication of THz Fibers and Waveguides. J. Appl. Phys..

[18] Koral C., Mazaheri Z., Andreone A. (2023). A Large Area Wide Bandwidth THz Phase Shifter Plate for High Intensity Field Applications. Photonics.

[19] Gong H., Huang J., Wang J., Zhao P., Guo M., Liang C., Bai D., Jiang Z., Li R. (2024). Additive Manufacturing for Terahertz Metamaterials on the Dielectric Surface Based on Optimized Electrohydrodynamic Drop-on-Demand Printing Technology. ACS Appl. Mater. Interfaces.

[20] Wu G.-B., Chen J., Yang C., Chan K.F., Chen M.K., Tsai D.P., Chan C.H. (2024). 3-D-Printed Terahertz Metalenses for Next-Generation Communication and Imaging Applications. Proc. IEEE.

[21] Carvalho S.S., Reis J.R.V., Mateus A., Caldeirinha R.F.S. (2024). Exploring Design Approaches for 3D Printed Antennas. IEEE Access.

[22] Shrotri A., Mukherjee A.K., Stübbe O., Preu S. THz-Characterization of Additively Manufactured Spiral Shaped Waveguides. Proceedings of the 2023 IEEE 11th Asia-Pacific Conference on Antennas and Propagation (APCAP).

[23] Lu Y., Zhu H., Zaman A.M., Rennie A.E.W., Lin H., Tian Y., Degl’Innocenti R. (2023). Contactless 3D Surface Characterization of Additive Manufactured Metallic Components Using Terahertz Time-Domain Spectroscopy. Opt. Mater. Express.

[24] Perraud J.B., Obaton A.F., Bou-Sleiman J., Recur B., Balacey H., Darracq F., Guillet J.P., Mounaix P. (2016). Terahertz Imaging and Tomography as Efficient Instruments for Testing Polymer Additive Manufacturing Objects. Appl. Opt..

[25] Busboom I., Nguyen T.T., Christmann S., Feige V.K.S., Haehnel H., Tibken B. Terahertz Imaging of 3D Print Infill Structures. Proceedings of the 2021 15th European Conference on Antennas and Propagation (EuCAP).

[26] (2017). Geometrical Product Specifications (GPS)—Geometrical Tolerancing—Tolerances of Form, Orientation, Location and Run-Out.

[27] Karaliūnas M., Nasser K.E., Urbanowicz A., Kašalynas I., Bražinskienė D., Asadauskas S., Valušis G. (2018). Non-Destructive Inspection of Food and Technical Oils by Terahertz Spectroscopy. Sci. Rep..

[28] Recur B., Younus A., Salort S., Mounaix P., Chassagne B., Desbarats P., Caumes J.-P., Abraham E. (2011). Investigation on Reconstruction Methods Applied to 3D Terahertz Computed Tomography. Opt. Express.

[29] Zhao Z., Alzubaidi L., Zhang J., Duan Y., Gu Y. (2024). A Comparison Review of Transfer Learning and Self-Supervised Learning: Definitions, Applications, Advantages and Limitations. Expert Syst. Appl..

[30] Brahm A., Kunz M., Riehemann S., Notni G., Tünnermann A. (2010). Volumetric Spectral Analysis of Materials Using Terahertz-Tomography Techniques. Appl. Phys. B.

[31] Wang S., Mei H., Liu J., Chen D., Wang L. (2022). A Terahertz Identification Method for Internal Interface Structures of Polymers Based on the Long Short-Term Memory Classification Network. Polymers.

[32] Hung Y.-C., Su W.-T., Chao T.-H., Lin C.-W., Yang S.-H. (2024). Terahertz Deep Learning Fusion Computed Tomography. Opt. Express.

[33] Wang T., Wang C., Wang Z., Zou K., Zhou Y., Shen S., Liang B., Zhang M., Wang X., Wang K. (2024). Metal Artifact Suppression in Terahertz Computed Tomography. Opt. Express.

